# Reconstruction of an Unguarded Tricuspid Orifice Using a Simplified Sliding Plasty Technique

**DOI:** 10.1093/icvts/ivag087

**Published:** 2026-03-24

**Authors:** Maurits Zegel, Elke S Hoendermis, Joost P van Melle, Ryan E Accord

**Affiliations:** Department of Pediatric and Congenital Cardiothoracic Surgery, Center for Congenital Heart Disease, University Medical Center Groningen, University of Groningen, 9700 RB Groningen, The Netherlands; Department of Cardiology, Center for Congenital Heart Disease, University Medical Center Groningen, University of Groningen, 9700 RB Groningen, The Netherlands; Department of Cardiology, Center for Congenital Heart Disease, University Medical Center Groningen, University of Groningen, 9700 RB Groningen, The Netherlands; Department of Pediatric and Congenital Cardiothoracic Surgery, Center for Congenital Heart Disease, University Medical Center Groningen, University of Groningen, 9700 RB Groningen, The Netherlands

**Keywords:** unguarded tricuspid orifice, congenital cardiac surgery, tricuspid valve abnormalities, tricuspid regurgitation, Adult Congenital Heart Disease, right heart pathology

## Abstract

Unguarded tricuspid orifice is a rare anomaly of the tricuspid valve characterized by complete absence of tricuspid valve tissue and chordae on a proportion of the annulus. Management strategies vary widely. We report a case of successful repair of an unguarded tricuspid orifice with a simplified technique.

A 35-year-old male presented with severe tricuspid regurgitation and right ventricular volume overload. Intraoperative inspection of the valve revealed an unguarded tricuspid orifice. For repair, sliding plasty of the anterior and posterior leaflets was performed, followed by ring annuloplasty and commissuroplasty. Postoperative, echocardiogram showed minimal residual tricuspid regurgitation and significantly improved right ventricular dimensions.

This case highlights the possibility of successful repair of an unguarded tricuspid orifice. If feasible, repair can be a good choice.

## INTRODUCTION

Unguarded tricuspid orifice is a congenital malformation where a normal orifice between the right atrium and ventricle exists, with complete absence of tricuspid valve tissue and chordae on a proportion of the annulus. Unguarded tricuspid orifice was first described in 1964. Unguarded tricuspid orifice is associated with pulmonary atresia and an intact ventricular septum.^1^ Natural history ranges from fetal death to asymptomatic survival into adulthood. When symptoms or right ventricular dysfunction occur, intervention should be considered. The purpose of this case report is to describe the diagnostic process and successful repair of unguarded tricuspid orifice.

## CASE REPORT

The case involves a 35-year-old male who recently immigrated from Egypt to the Netherlands. His extensive medical history included human immunodeficiency virus (HIV), latent tuberculosis, hepatitis C, and systemic lupus erythematosus (SLE) with lung and kidney involvement. The SLE was complicated by septic arthritis of the right hip, for which surgical intervention was required. Over the past decades, SLE and HIV have been well-managed. The patient was referred for an enlarged cardiac silhouette on X-ray and right bundle branch block on ECG. Upon inquiry, the patient reported shortness of breath during moderate exercise.

Echocardiography showed a left ventricular ejection fraction of 50%, significant diastolic flattening of the interventricular septum, a dilated right atrium, dilatated tricuspid annulus of 61 mm with torrential tricuspid regurgitation (vena contracta 24 mm) with backflow into the hepatic veins, right ventricle peak pressure of 15 mmHg and a dilated and volume overloaded right ventricle with reduced function. There were no signs of Ebstein anomaly. There was no pulmonary valve dysfunction. In the parasternal inflow views, only a small portion of the anterior leaflet was visible, suggesting a primary tricuspid regurgitation. Secondary causes like pulmonary hypertension and intracardial shunting were excluded by heart catheterization. MRI revealed a volume-overloaded right ventricle with severe tricuspid regurgitation without any findings supporting a morphologic anomaly of the tricuspid valve. SLE and endocarditis were considered but ruled out by other diagnostic investigations. Surgical intervention became imperative due to severe tricuspid regurgitation with symptomatic right heart failure, although the mechanism of the regurgitation was not yet fully understood.

**Figure 1.**
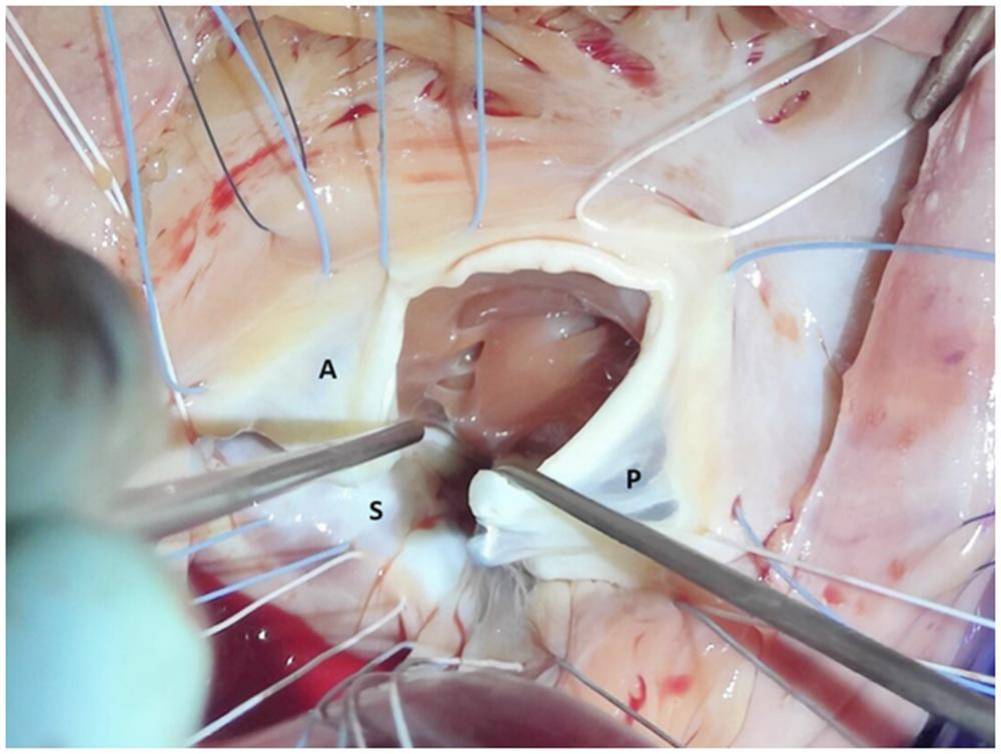
Operative view showing an unguarded tricuspid orifice between the anterior (A) and posterior (P) leaflets. (S) = septal leaflet of the tricuspid valve.

**Figure 2.**
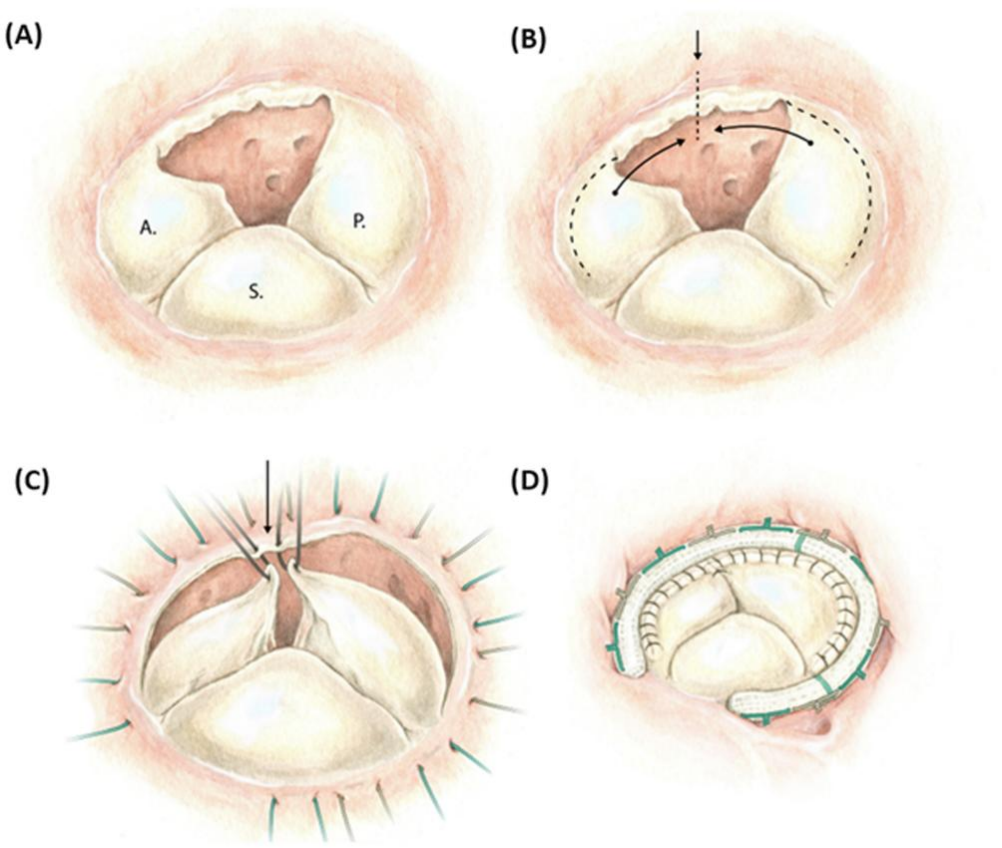
Surgical repair technique of an Unguarded tricuspid orifice: (A) Unguarded tricuspid orifice visible between the anterior (A) and posterior (P) leaflet, Next to septal (S) leaflet. (B and C) subtotal detachment of a nterior and posterior leaflets, followed by sliding plasty. (D) Reduction annuloplasty and additional commissural stitch, resulting in a competent valve

Surgery was performed through sternotomy. Extracorporeal circulation was initiated after aortic and bicaval cannulation. Examination of the tricuspid valve showed an incompletely developed anterior tricuspid leaflet, where a limited edge of the valve was present without any subvalvular apparatus. The remaining anatomy of the tricuspid valve showed a normal position of the annulus with structurally intact septal and posterior leaflets, as well as their subvalvular apparatus (Figure 1). The finding of an unguarded tricuspid orifice was unexpected. The valve was judged as repairable. The anterior and posterior leaflets were subtotally detached from the annulus insertion. These segments were repositioned towards each other over the unguarded proportion of the tricuspid annulus, resulting in the leaflets lying adjacent to each other. Reduction annuloplasty was performed by implanting a 36 mm tricuspid ring. An additional commissuroplasty was placed between the now lying adjacent anterior leaflet and posterior leaflet (Figure 2).

After weaning extracorporeal circulation, the echocardiogram revealed minimal residual tricuspid regurgitation with improvement in right ventricular function. Six days after surgery, the patient was discharged from the hospital in good condition. At 3 months follow-up, echocardiography demonstrated trivial tricuspid regurgitation without stenosis, with a peak pressure gradient of 1.2 mmHg, a decreased right ventricular end-diastolic diameter from 70 to 43 mm, and improved right ventricular function (Video 1).

## DISCUSSION

Unguarded tricuspid orifice is a rare congenital anomaly, and its optimal management remains uncertain due to the limited number of reported cases. The natural course depends on the presence of associated pathology and the extent of the affected region. Interventions for severe tricuspid regurgitation are recommended in symptomatic patients, in case of severe right ventricular dilatation or decrease of right ventricular function.^2^ Generally, if surgical intervention of a dysfunctional tricuspid valve is indicated, repair of the tricuspid valve should be preferred over replacement due to superior perioperative, midterm, and event-free survival outcomes.^2^ However, repairing an unguarded tricuspid orifice can be challenging. Different strategies to treat unguarded tricuspid orifice have been described, ranging from conservative treatment to tricuspid valve replacement to the Fontan procedure with total right ventricular exclusion.^3^ Only one other repair of an unguarded tricuspid orifice has been reported, using complex reconstructive techniques, including approximation of the anterior papillary muscle to the ventricular septum and closure of the posterior orifice.^4^ In other forms of severe valve dysplasia, such as Ebstein anomaly, patch augmentation using right atrial wall tissue has been described, offering viable autologous tissue for reconstruction.^5^ In our case, however, annular reduction provided sufficient native valve tissue to permit reconstruction without patch augmentation.

This case demonstrates that an unguarded tricuspid orifice can be successfully repaired using a simplified sliding plasty technique, offering a practical and effective alternative in anatomically suitable patients.

## Data Availability

All data are available on request.
